# Supplementary material for: Arabidopsis Actin-Binding Protein WLIM2A Links PAMP-Triggered Immunity and Cytoskeleton Organization
Source: Int J Mol Sci. 2024 Oct 30;25(21):11642. doi: 10.3390/ijms252111642 (PMC11545931; doi:10.3390/ijms252111642)
Supplement: Supplementary file 1 [file ijms-25-11642-s001.zip › ijms-3237218-Supplementary Figures.pdf]

Supplementary Information

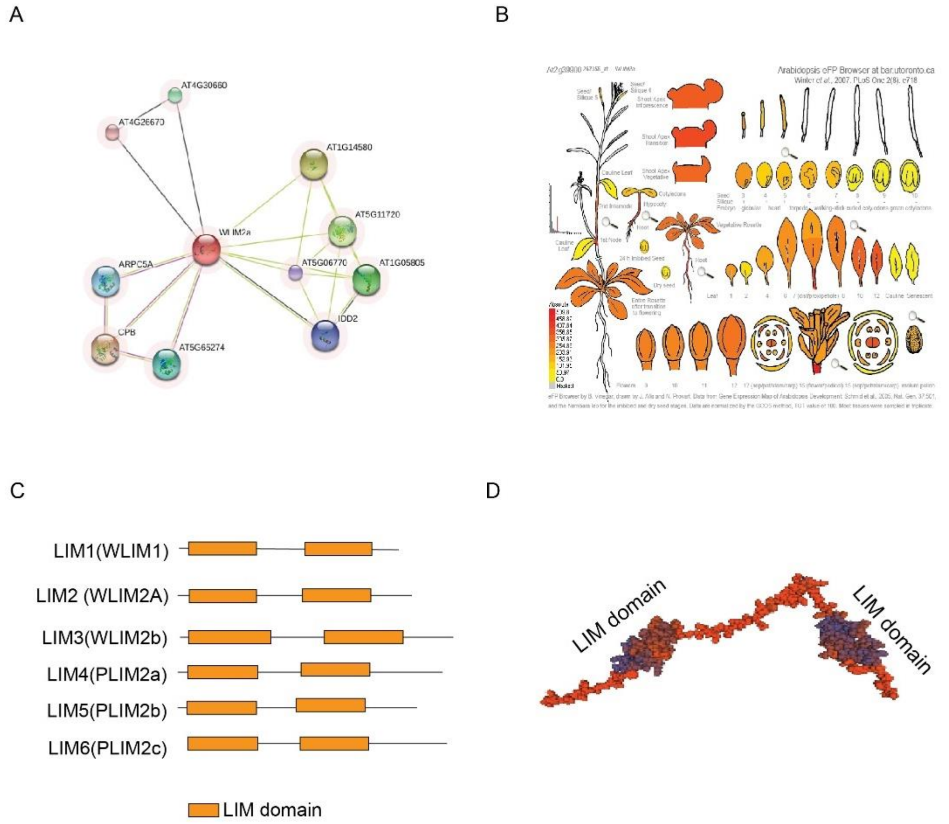

Figure S1. (**A**) Interaction networks from the STRING website, showing results of the potential partners for the WLIM2A protein. (**B**) eFP browser view of gene expression during *Arabidopsis* development. Expression of the WLIM2A, At2g39900, showing stronger expression widely seen in vegetative and reproductive stages. Expression strength coded by color: yellow=low, red=high. The *Arabidopsis* eFP Browser is located at bar.utoronto.ca, published in Winter et al., 2007 (**C**) Schematic comparison of the domain architecture of the *AtLIM* gene family. (**D**) Predicted protein model of WLIM2A build from Swiss-model (Uniprot Id: O04193). The target sequence was performed against the SWISSMODEL template library. Two LIM domain contains double zinc finger motif at 8-68th and 107-167th position, respectively. Orange- full protein, blue and orange- LIM domain.

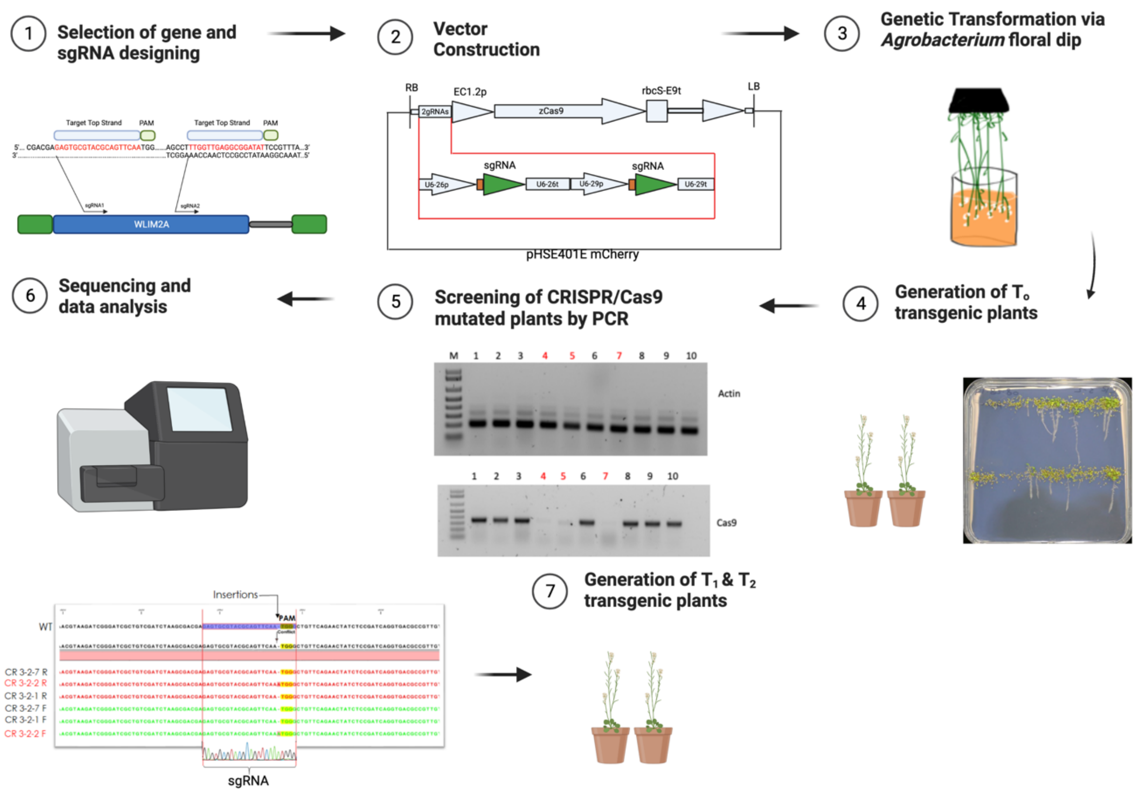

Figure S2. Workflow and Generation of CRISPR/Cas9 knockout mutant lines in *Arabidopsis*. Physical map of PHSE401 mcherry vector carrying two-gRNAs targeting two regions of *Arabidopsis* gene *WLIM2A.* The construct was transformed via *Agrobacterium* floral dip method. Screening of T1 transgenic plants on agar plates and selected the survival to grow in the soil. The DNA of plants was extracted, PCR amplified, and sequenced. Analysis of mutations using sequence alignment software to select for Indels and select homozygous mutants. Screening for T2 and T3 homozygous mutants and cas9 free lines is a key-steps to generate CRISPR/Cas9 lines.

Phosphopeptide	Phosphomotif	mpk3-F	mpk4-F	mpk6-F	WT-F	mpk3-M	mpk4-M	mpk6-M	WT-M
**(t)*PSRLAGMFSGTQDK**	T*P or RxxS*	3.75E+06	3.99E+06	4.41E+06	5.13E+06	2.91E+06	2.76E+06	2.09E+06	1.90E+06

Figure S3. Table showing the relative abundance of the phosphopeptides. The abundance was measured in WT and mapk mutants after treatment by flg22 (Rayapuram et al., 2018).

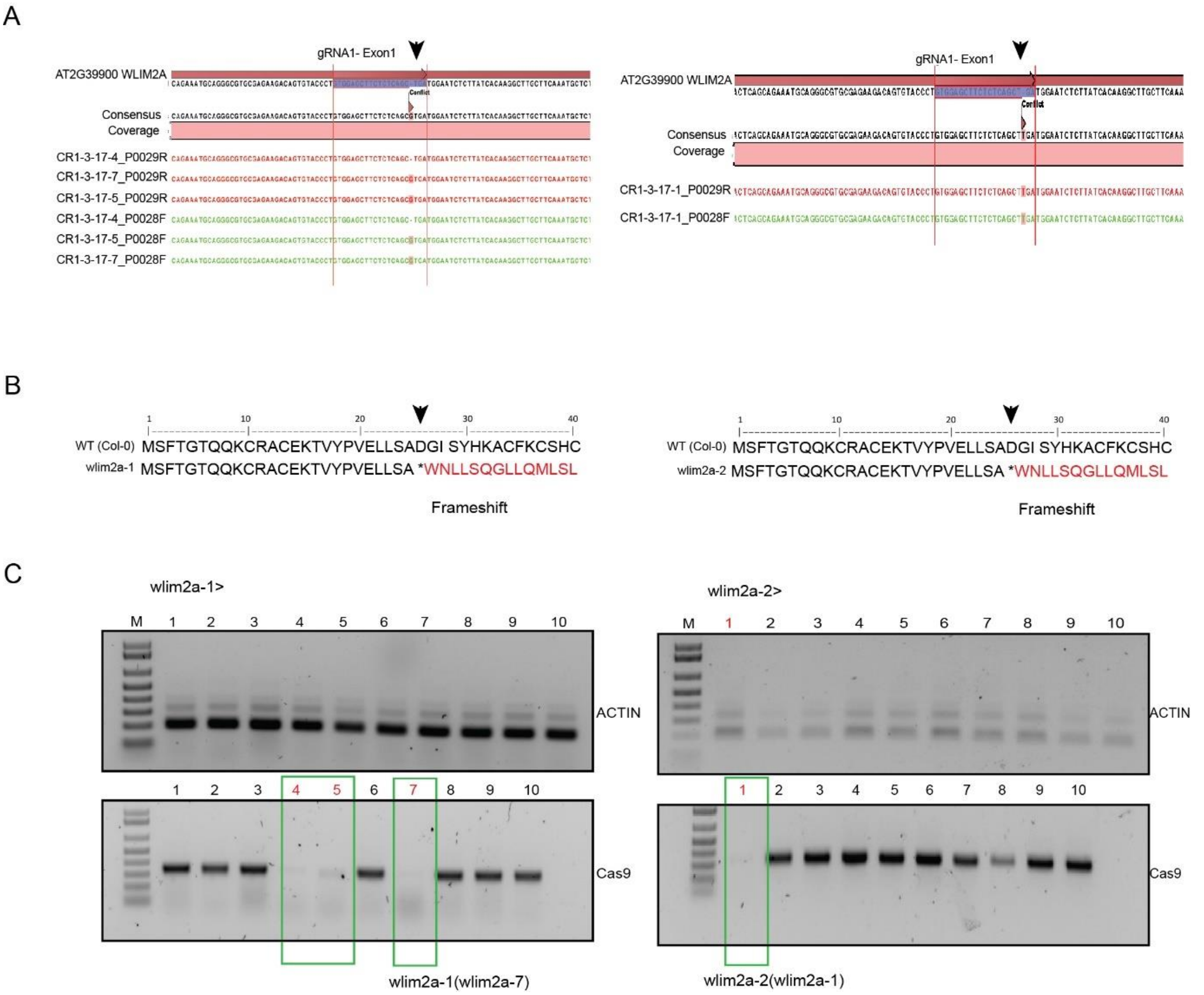

Figure S4. Inheritance of CRISPR/Cas9 mutations. (**A**) *In silico* analysis of nucleotide sequence of the targeted locus for Col-0 and *wlim2a*. One bp upstream of the PAM (TGA) within the sgRNA target sequence (highlighted), Cas9 cut site is indicated with a triangle (**B**) Sequence alignment of the targeted locus for Col-0, *wlim2a-1*, and *wlim2a-2*, all causing frameshift and formation of premature stop codon showing the frameshift indel resulting in a truncated protein of *wlim2a-1*, and *wlim2a-2.* (**C**) PCR amplification of Cas9 free CRISPR/Cas9 lines in T2 seedlings from the *wlim2a-1*(1-10), and *wlim2a-2* (1-10). Green boxed plants were Cas9 free lines.

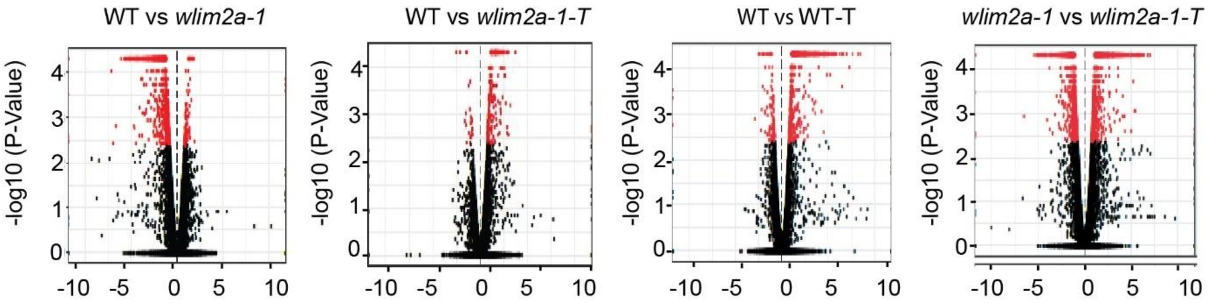

Figure S5. A comparison between mock and treated samples of WT and *wlim2a-1* can be seen in the volcano plots. A plot is shown of log2 fold change against log10 (P-value).

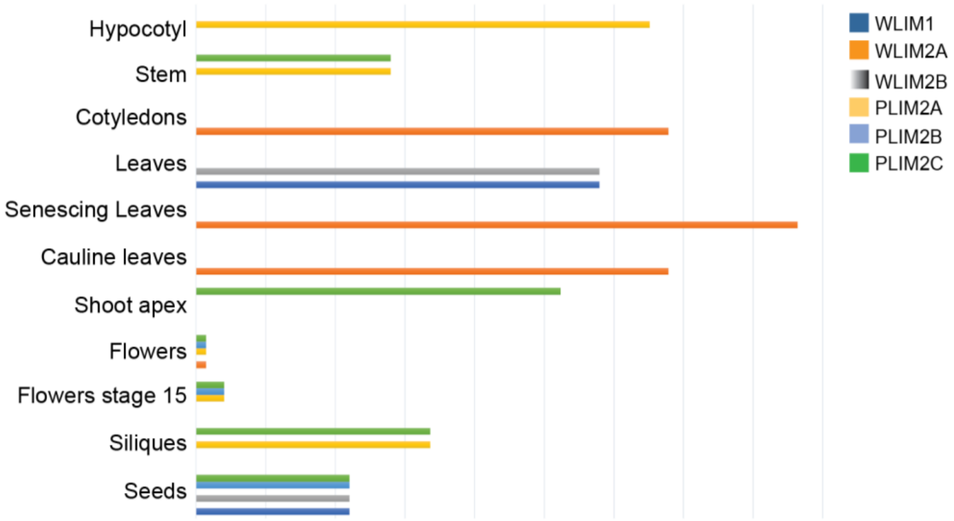

Figure S6. Microarray analysis of the LIM gene expression in *Arabidopsis*. (**A**) Electronic RNA gel blot analysis of LIM gene expression in different organs and development stages using the microarray. The data is retrieved from the AtGenExpress Schmid et al., 2005.

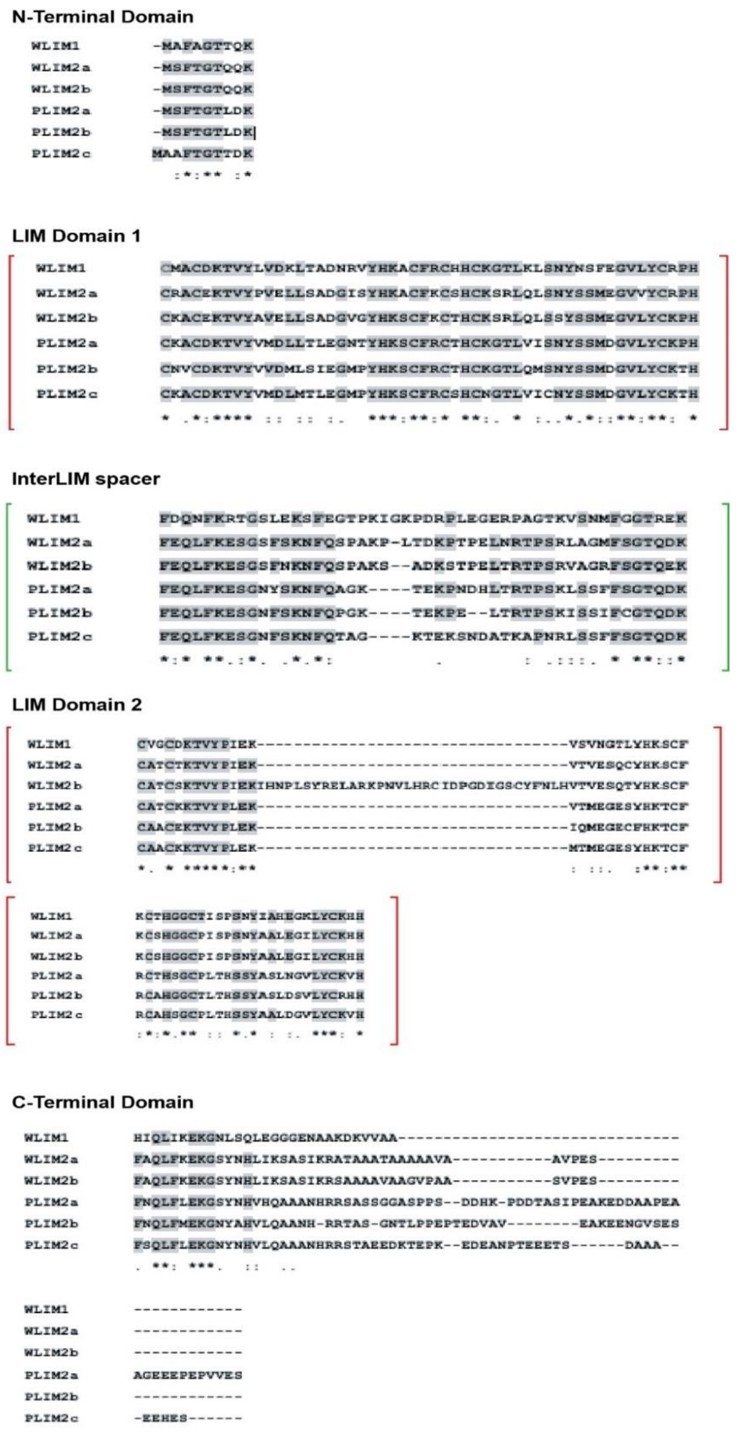

Figure S7. Multiple sequence alignment of *Arabidopsis* LIM proteins. Grey Shading indicates amino acids residues that are identical. The sequences were aligned using the Clustal Omega program(https://www.ebI.ac.uk/Tools/ms a/clustalo/).The functional domains were identified through Uniprot (https://www.uniprot.org/uniprotkb/O04193/entry) and Pfam database (https://www.ebi.ac.uk/interpro/protein/UniProt/O04 193/entry/pfam/#table). The LIM domain and interLIMspacer are indicated in red and green, respectively.
